# Algae-Derived Anti-Inflammatory Compounds against Particulate Matters-Induced Respiratory Diseases: A Systematic Review

**DOI:** 10.3390/md19060317

**Published:** 2021-05-30

**Authors:** Pek Xyen Tan, Krishnapriya Thiyagarasaiyar, Cheng-Yau Tan, You-Jin Jeon, Mohd Shahrul Mohd Nadzir, Yong-Jiang Wu, Liang-Ee Low, Atanas G. Atanasov, Long Chiau Ming, Kai Bin Liew, Bey-Hing Goh, Yoon-Yen Yow

**Affiliations:** 1 Department of Biological Sciences, School of Medical and Life Sciences, Sunway University, Subang Jaya 47500, Malaysia; pek.t6@imail.sunway.edu.my (P.X.T.); krishna.t@imail.sunway.edu.my (K.T.); cheng.t20@imail.sunway.edu.my (C.-Y.T.); 2 Department of Marine Life Sciences, Jeju National University, Jeju 63243, Korea; youjinj@jejunu.ac.kr; 3 Department of Earth Sciences and Environmental, Faculty of Science and Technology, Universiti Kebangsaan Malaysia, Bangi 43600, Malaysia; shahrulnadzir@ukm.edu.my; 4 College of Pharmaceutical Sciences, Zhejiang University, Hangzhou 310058, China; yjwu@zju.edu.cn (Y.-J.W.); lowliangee@gmail.com (L.-E.L.); 5 Biofunctional Molecule Exploratory (BMEX) Research Group, School of Pharmacy, Monash University Malaysia, Subang Jaya 47500, Malaysia; 6 Ludwig Boltzmann Institute of Digital Health and Patient Safety, Medical University of Vienna, 1090 Vienna, Austria; Atanas.Atanasov@dhps.lbg.ac.at; 7 Institute of Genetics and Animal Biotechnology of the Polish Academy of Sciences, 05-552 Magdalenka, Poland; 8 Department of Pharmacognosy, University of Vienna, 1090 Vienna, Austria; 9 PAP Rashidah Sa’adatul Bolkiah Institute of Health Sciences, Universiti Brunei Darussalam, Gadong BE1410, Brunei; longchiauming@gmail.com; 10 Faculty of Pharmacy, University of Cyberjaya, Cyberjaya 63000, Malaysia; liewkaia@yahoo.com

**Keywords:** algae, particulate matter, anti-inflammation, respiratory diseases

## Abstract

Air pollution has recently become a subject of increasing concern in many parts of the world. The World Health Organization (WHO) estimated that nearly 4.2 million early deaths are due to exposure to fine particles in polluted air, which causes multiple respiratory diseases. Algae, as a natural product, can be an alternative treatment due to potential biofunctional properties and advantages. This systematic review aims to summarize and evaluate the evidence of metabolites derived from algae as potential anti-inflammatory agents against respiratory disorders induced by atmospheric particulate matter (PM). Databases such as Scopus, Web of Science, and PubMed were systematically searched for relevant published full articles from 2016 to 2020. The main key search terms were limited to “algae”, “anti-inflammation”, and “air pollutant”. The search activity resulted in the retrieval of a total of 36 publications. Nine publications are eligible for inclusion in this systematic review. A total of four brown algae (*Ecklonia cava*, *Ishige okamurae*, *Sargassum binderi* and *Sargassum horneri*) with phytosterol, polysaccharides and polyphenols were reported in the nine studies. The review sheds light on the pathways of particulate matter travelling into respiratory systems and causing inflammation, and on the mechanisms of actions of algae in inhibiting inflammation. Limitations and future directions are also discussed. More research is needed to investigate the potential of algae as anti-inflammatory agents against PM in in vivo and in vitro experimental models, as well as clinically.

## 1. Introduction

Particulate matter (PM) is the term used to describe a mixture of microscopic particles of solid and liquid matter found in the air [1]. Air pollution due to high levels of PM in the air is leading to various environmental issues and has global health implications, particularly in the area of public health. According to the World Health Organization (WHO), nearly 4.2 million early deaths are related to ambient air pollution [2]. Unfortunately, this emerging air pollution problem remains unsolved due to various reasons spanning from economical concern to geopolitical interest.

Generally, human activities such as open burning, emissions from factories and vehicles, rapid urbanization, development of the industrial sector, and coal-burning power plants are deemed as major causes of air pollution. In the South-East Asia (SEA) region, high concentrations of suspended PM can cause the haze phenomenon, which significantly affects health, crop production, the economy and much more [3]. There are a variety of PM categories, of which some are based on aerodynamic diameter: coarse particles PM_10_ (2.5–10 µm), fine particles PM_2.5_ (<2.5 µm), and ultrafine particles PM_0.1_ (<0.1 µm or 100 nm); these are considered harmful pollutants. PM_10_ can deposit in the upper respiratory tract, whereas PM_2.5_ is primarily produced from the emission of combustion sources and it can be more easily inhaled compared to PM_10_. They readily reach the alveoli of the lungs and affect the pulmonary and systematic circulation, while PM_0.1_ is primarily derived from vehicle emissions and can migrate from alveoli to the circulatory system [4].

Inhalation of high concentrations of particulate pollution may adversely impact human organs and body systems, especially the respiratory system, which might lead to different diseases, such as asthma, chronic obstructive pulmonary disease (COPD), lung cancer and respiratory infections, and even combine with stroke and heart diseases [5]. As studied by Jiang, Mei, and Feng [6], individuals with asthma and COPD are vulnerable to the harmful consequence of air pollutants, which may lead to an increase in respiratory mortality and morbidity. Symptoms such as allergic reactions, wheezing, coughing, breathing problems, and inflammation responses in macrophage cells are found to be associated with exposure to air pollutants [7,8]. Hence, inhibition of inflammation in the lungs is crucial. Therefore, there is an urgent need to search for effective and safe anti-inflammatory treatments that might prevent the relentless progression of respiratory diseases.

The search for novel treatments has led to the exploration of natural marine products as anti-inflammatory agents for treating inflammatory diseases such as respiratory diseases. Unlike terrestrial plants, algae have rapid growth rates and do not require arable land and fresh water for cultivation [9]. As algae sometimes grow in harsh conditions and are exposed to biotic and abiotic stresses, they possess unique metabolic pathways to produce secondary metabolites with biofunctional properties that might aid their survival [10]. Considering their advantages and therapeutic properties, algae have been a focus and actively explored for their potential nutraceuticals and pharmacological applications in recent years.

Previous studies reported that algae have anti-inflammatory properties due to the presence of bioactive metabolites [11,12,13,14]. However, there is a relatively low number of studies conducted specifically on anti-inflammatory activity against air pollutants, which is considered to be a unique area with great potential for application. Various seaweed derived metabolites, such as fucosterol [15], alginic acid [16], dieckol [17], eckol [17], diphlorethohydroxycarmalol [18], gallic acid [19], mojabanchromanol [20], phlorotannins and flavonoid [21,22], have been proven to exhibit anti-inflammatory activity against air pollutants. Hence, this review aims to summarize and evaluate the evidence of the anti-inflammatory activity of algae-derived metabolites between 2016 and 2020, to examine if these metabolites can be applied to tackle air pollutant-induced respiratory diseases. The underlying mechanisms are also included. Furthermore, the challenges, limitations, and suggestions for future research are discussed in this review.

## 2. Results

**Figure 1:**
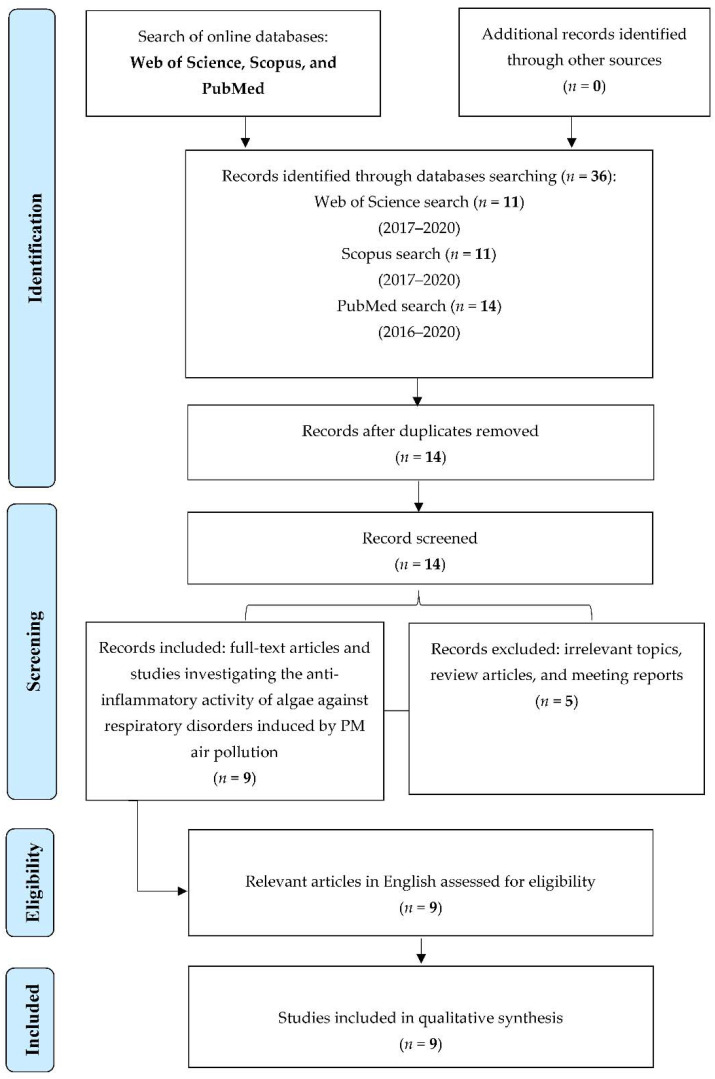
Flowchart of the selection of studies.

In total, 36 articles were identified from the electronic databases. The Web of Science search identified 11 articles published between 2017 and 2020, as did the Scopus search; the PubMed search, however, identified 14 published articles between 2016 and 2020. After removing the 22 duplications, the remaining 14 articles were reviewed. Firstly, each article was reviewed to ensure that they were relevant to the topic. Five articles were removed, including one meeting report, two review articles, and two irrelevant articles. The remaining several articles were retrieved for further review to provide more information on the current systematic review. A final of nine articles were included in the qualitative synthesis (Figure 1).

## 3. Discussion

Increasing evidence has shown that exposure to PM leads to adverse health impacts, especially respiratory diseases via epigenetic mechanisms [5,6,8,23]. Epigenetic effects of PM include alteration of epigenetic marks, which in turn changes related gene expression without changing the DNA sequence. The arisen oxidative stress triggers inflammatory pathways discussed in detail below. Studies have indicated that the groups vulnerable to PM exposure are the prenatal and early life stages, where the beneficial epigenetic effects induced from nutrition are critically important as a preventive measure against long-term health risks [5,23]. For example, perinatal supplementation with olive oil containing polyphenols, fish oil containing omega-3 polyunsaturated fatty acids, and a high-fiber diet producing microbial short chain fatty acids can induce immunomodulatory and anti-inflammatory responses in airway allergic diseases [23,24,25,26]. Algae, which produce a wide variety of bioactive compounds, have also demonstrated potential in protection against PM-induced respiratory disorders (Table 1).

### 3.1. Pathways of Particulate Matter Travelling into the Respiratory System and Causing Inflammation

Ambient PM enters the respiratory system through inhalation and travels into the alveolar sacs to induce inflammation. PM such as PM_2.5_ contains polycyclic aromatic hydrocarbons (PAHs) that can stimulate inflammation in human bronchial epithelial cells [27]. When PAHs pass through the epithelium of alveoli, it can cause oxidative stress, activation of neutrophils, and mitochondrial damage. PM-induced oxidative stress may initiate intracellular ROS generation [28] and lipid peroxidation [29], whereby the nuclear factor erythroid 2-related factor 2/heme oxygenase-1 (NRf2/HO-1) pathway will be affected, resulting in the alteration of antioxidant enzyme transcription. The uncontrolled activation of polymorphonuclear neutrophils may increase the inflammatory responses, leading to tissue damage via oxidative stress and producing ROS [21]. It also enhances the inflammatory responses through the ROS-mediated activation of nuclear factor-kappa B (NF-κB) and MAPKs pathways.

**Figure 2:**
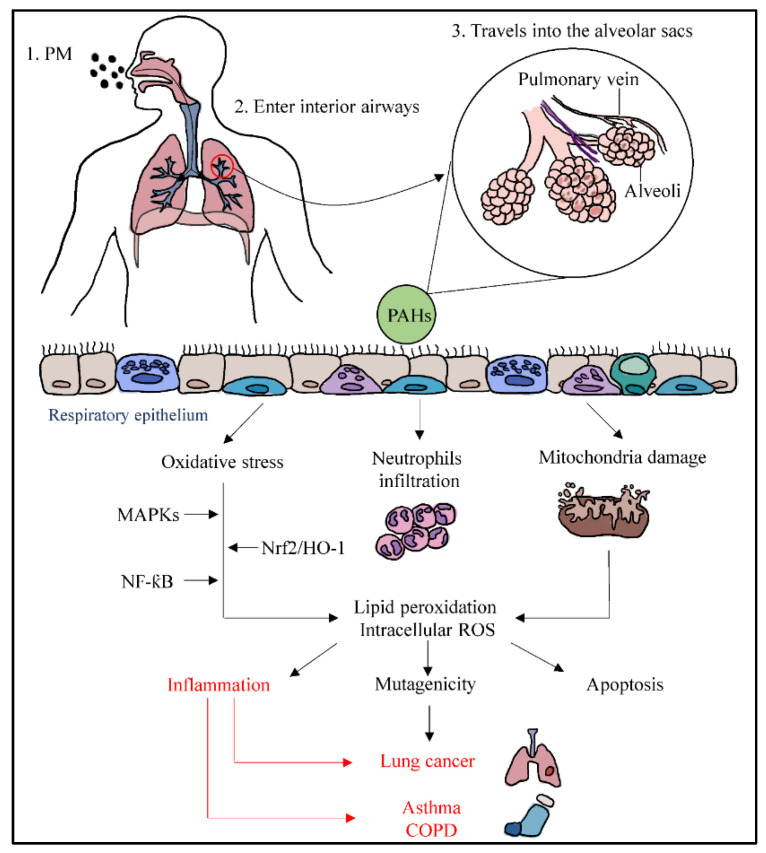
Pathways of particulate matter depositing into the respiratory system through inhalation, resulting in systemic lung inflammation, lung cancer, asthma, and COPD.

The potential damage of mitochondria transmembrane caused by PM associated with ROS is another mechanism which may lead to mutagenicity and apoptosis. Mitochondria are the main target of ROS, and they lack nucleus and protective histones, which makes them specifically susceptible to ROS-stimulated destruction [30]. ROS can break the DNA strands when there is a failure of ROS catalyzation. This results in alterations in gene expression profiles and cellular biochemical profiles, leading to cell apoptosis, and remodeled cell function may be triggered by the responses of chromosome damage from PM stimulation [27]. This epigenetic and abnormal alteration could lead to lung cancer. On the other hand, lipid peroxidation can disrupt the double membrane of mitochondria and uncouple its membrane-bound complex [30]. When inflammation caused by oxidative stress is uncontrolled, PM can trigger inflammatory effects and cause changes in the lungs, associated with the elevated exacerbation of chronic respiratory conditions such as asthma and COPD (Figure 2).

### 3.2. Algae Metabolites with Anti-Inflammatory Activity against Air Pollutants

**Figure 3:**
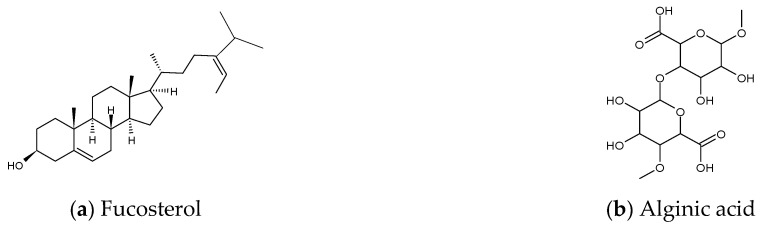

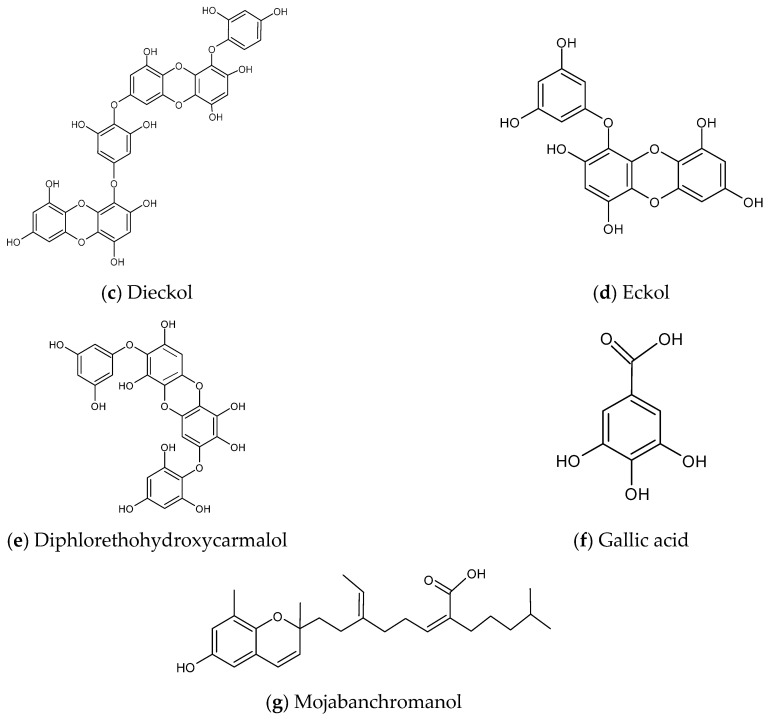
Chemical structures of metabolites derived from algae with anti-inflammatory effects against air pollutants. (**a**) Fucosterol, (**b**) Alginic acid, (**c**) Dieckol, (**d**) Eckol, (**e**) Diphlorethohydroxycarmalol, (**f**) Gallic acid, (**g**) Mojabanchromanol.

According to the nine selected studies [7,15,16,17,18,19,20,21,22], only four brown algae, namely *Ecklonia cava*, *Ishige okamurae*, *Sargassum binderi* and *Sargassum horneri* from Korea, have been explored with respect to anti-inflammatory activity against air pollutants. The prominent secondary metabolite groups from these four brown algae with anti-inflammatory effects against PM are classified into phytosterol, polysaccharides, and polyphenols; furthermore, the ethanol extract complex of *Sargassum horneri* also exhibits similar anti-inflammatory effect. No studies have been reported on metabolites from red or green algae in this aspect. Table 1 summarizes anti-inflammatory properties of algae-derived metabolites and ethanol extracts against air pollutants. The chemical structures of some prominent metabolites are shown in Figure 3.

#### 3.2.1. Phytosterols

Phytosterols are a group of the main compounds of lipids found in marine algae and have various beneficial health effects [31]. They are natural constituents of plant cell membranes with a similarity to cholesterol structure, but with minor differences in the position of ethyl and methyl groups [32]. Fucosterols are the most commonly found phytosterols in brown algae [33].

##### Fucosterol

Fucosterol from *S. binderi* is the most abundant phytosterol which has been proven to exhibit anti-inflammatory effects against damage induced by PM on the A549 immortalized alveolar basal epithelial cells [15]. It is mainly found to act through reducing the DNA damage on a cellular level, as observed from a decrease in the Sub-G1 cell population. As inflammation leads to oxidative stress-stimulated apoptosis, fucosterol significantly decreases the upregulation of inflammatory mediators (cyclooxygenase-2 (COX-2), prostaglandin E2 (PGE2), and inducible nitric oxide synthase (iNOS)) and pro-inflammatory cytokines (tumor necrosis factor-alpha (TNF-α), interleukin-6 (IL-6), and interleukin-beta (IL-1β)) expression by inhibiting the NF-κB and MAPKs pathways in a dose-dependent manner (12.5–50 µg/mL). This indicates that the expression levels of molecule mediators, such as IL-6 and TNF-α, could aid as a biomarker to assess the severity of fine dust (FD) exposure. FD consists of tiny particles with an aerodynamic diameter equal to or less than 10 µm [34]. The release of TNF-α and IL-6, the pro-inflammatory cytokines, increases chemotactic activity for neutrophils. Additionally, IL-1β is induced by PAH, and nickel in PM can further enhance mucin secretion, thicken airways, cause inflammation with macrophages and neutrophils, and develop symptoms into respiratory diseases such as COPD and asthma [19].

#### 3.2.2. Polysaccharides

Polysaccharides are considered to be the largest group of compounds with biological activities in algae—approximately 60% of the metabolites [35]. They are water-soluble compounds with hydrophilic properties, and they have a regular structure that varies from linear to highly branched. They are formed by chains of monosaccharide units that connect via glycosidic bonds. Examples of the main compounds of polysaccharides include alginates, fucoidans, ulvans, carrageenans, and laminarans.

Alginic acid, a non-sulphated polysaccharide from brown alga *S. horneri* (SHA), when tested against the FD-stimulated inflammatory responses in HaCaT (human keratinocytes) and RAW264.7 mouse macrophages, showed anti-inflammatory effects against PM [16]. Evidently, the PAHs in PM and PM-derived ROS contribute to inflammation [27]. The upstream activations of NF-κB and MAPKs were measured to evaluate the inflammation caused by PAHs. SHA treatment had significantly increased the cell viability, and decreased the levels of pro-inflammatory cytokines, inflammatory mediators and intracellular ROS; inhibition of the NF-κB and MAPKs pathways was also observed.

#### 3.2.3. Polyphenols

Polyphenolic compounds, also known as phenolics, are typically isolated from brown algae. They differ from simple molecules, including phenolic acids and other simple polyphenolic compounds, as well as more complex compounds such as phlorotannins, which are made up of phloroglucinol (1,3,5-trihydroxybenzene) units of polymeric structures [36]. Main compounds belonging to the group of polyphenolic compounds include eckols, fucols, fuhalols, phlorethols, fucophlorethols, bromophenols, terpenoids, phlorotannins and flavonoid.

##### Dieckol and Eckol

Sanjeewa et al. [17] proved that dieckol from brown alga *Ecklonia cava* showed anti-inflammatory potential against PM-induced damage on RAW264.7 macrophages. The dieckol effectively attenuated the levels of PGE2, NO, inflammatory mediators, and pro-inflammatory cytokines from the exposure of RAW264.7 to PM. Dieckol also protected the macrophages against cellular damage by decreasing the intracellular ROS through the activation of superoxide dismutase production, which leads to the Nrf2/HO-1 pathway. Nrf2/HO-1 are the essential antioxidant proteins that scavenge the production of NO through antioxidant mechanisms during the inflammation process [37]. Hence, dieckol can protect the cells against PM-stimulated inflammation and oxidative stress by inducing their anti-inflammatory and antioxidant pathways respectively.

##### Diphlorethohydroxycarmalol (DPHC)

Anti-inflammatory effects of DPHC from the brown alga *I. okamurae* in RAW264.7 macrophages, HaCaT cells and zebrafish embryos were investigated by Fernando et al. [18]. DPHC was proven to inhibit the production of PGE2, COX-2, IL-6 and IL-1β dose-dependently at concentrations of 6.25–25.00 µg/mL in HaCaT keratinocytes, and the expression of PGE2, NO, iNOS and TNF-α in macrophages. Furthermore, DPHC decreased the production of ROS and NO induced by FD in the zebrafish embryos, which consequently decreased cell death. Also, DPHC reduced the larval mortality and blocking of larval gills by FD. This indicates that the airway of the respiratory system (larval gills) of the in vivo experimental model will not be obstructed by FD after the DPHC treatment.

##### Phenolic Acid

Phenolic acid, particularly gallic acid from the brown alga *S. horneri* ethanol extract (SHE), showed anti-inflammatory effects against PM-induced type II alveolar epithelial cell lines, MLE-12 [19]. mRNA expression of toll-like receptors (TLRs) such as TLR2, TLR4 and TLR7, pro-inflammatory cytokines, and lung epithelial cell derived-chemokines (monocyte chemoattractant protein-1 (MCP-1), chemokine ligand 5 (CCL5) and IL-8) induced by PM were attenuated by the alga extract. The lung epithelial cell derived-chemokines are known to increase inflammatory responses, which can exacerbate asthmatic responses and lead to pulmonary sarcoidosis; thus, these chemokines should be reduced. mRNA expressions of IL-33 and pro-allergic cytokines thymic stromal lymphopoietin (TSLP) stimulated by PM were reduced as well. Additionally, the suppression of MAPK p38 phosphorylation by reducing tumor necrosis factor receptor-associated factor 6 (TRAF6) activation of proteins TNF and myeloid differentiation factor 88 (MyD88) was observed. PM-induced activation of the MAPK pathway in MLE-12 further suggested that it might be mediated through extracellular signal-regulated kinase (ERK) and c-Jun N-terminal kinase (JNK).

##### Chromene (Mojabanchromanol)

Chromenes result from the dehydration of chromanols. They are phenolic terpenoids with a ring of aliphatic and chromanol side chains. Herath et al. [20] proved that mojabanchromanol (a novel chromene) in SHE exhibited anti-asthmatic effects in BALB/c mice. It has the potential in treating PM-induced allergic asthma, which is an inflammatory respiratory disease. Mitigation of PM-induced dendritic cells can be observed. PM-stimulated eosinophil infiltration, which is believed to be the origin of Th2-mediated asthma, attenuated in the trachea, lung and bronchoalveolar lavage fluid (BALF) due to SHE. As an elevation of infiltrated inflammatory cells in BALF is the dominant biomarker in asthma pathogenesis, it should be reduced [20]. Besides, stimulation of mast cells infiltration in response to the activation of Th2 cells was reduced in the allergic lung tissues. SHE also reversed the activation of mast cells which went through degranulation, and the release of histamine and vasoactive mediators in the trachea. As IgE is involved in mast cells activation and degranulation, analysis of serum IgE levels further proved the reduction level by SHE. Furthermore, airway obstruction and mucus released from goblet cells exacerbating asthma could be observed, but these were successfully attenuated by SHE.

Moreover, CD4+ and CD8+ T cell populations were investigated. Similarly, SHE suppressed the PM-induced T cell populations. As SHE reduced the CD4+ T cells, it also decreased differentiation into Th2, whereas the reduced CD8+ T cells further attenuated the airway inflammation and hyperresponsiveness in PM-induced mice. Simultaneously, SHE further suppressed the Th17 cell response by reducing the mRNA expression of RAR-related orphan receptor gamma (RORγT) and phosphorylation of signal transducer and activator of transcription 3 (STAT3), which create the primary neutrophils for asthma. This led to the mitigation of neutrophil infiltration in the lung. mRNA expression of GATA3, the major transcription factor for Th2 cells differentiation, was also attenuated by SHE. PM-induced STAT5 translocation was another effect on transcription factors involved in the Th2 immune response, which was reduced by SHE. PM-stimulated IL-4, IL-5, IL-13 and IL-17a epithelial cell-derived cytokine expression was also reduced in asthmatic mice [20].

##### Phlorotannins and Flavonoid

The composition of metabolites such as phlorotannins and flavonoid from *S. horneri* ethanol extract (SHE) were proven to exhibit anti-inflammatory effects against PM in BALB/c mice models [21] and murine MH-S cells [22]. PM-induced NO secretion from the production of inflammatory mediators and pro-inflammatory cytokines was inhibited by phlorotannins and flavonoid from the SHE treatment. Besides, the metabolites from the SHE treatment induced Nrf2 and HO-1 activities, resulting in the upregulation of antioxidant genes and cytoprotective potential against oxidative stress induced by FD in BALB/c mice [21]. Furthermore, MAPKs [21,22] and NF-κB [22] pathways were inhibited by the metabolites. Specifically, the upregulated mRNA expression levels of the TLRs in PM-stimulated MH-S cells were inhibited by the metabolites, leading to the downstream activations of both pathways [22].

#### 3.2.4. Ethanol Extract

Besides bioactive compounds from algae, a relatively complex mixture derived from the solvent extraction method application on algae could also exhibit anti-inflammatory potential against the PM-induced damage. The protective effect of SHE against the FD-stimulated inflammation was investigated in RAW264.7 cells [7]. The production of inflammatory mediators and pro-inflammatory cytokines that stimulated NO production were inhibited by SHE treatment in the tested cells. Nrf2 and HO-1 activities were in-duced by SHE treatment, resulting in the upregulation of antioxidant genes and cytopro-tective potential against oxidative stress induced by FD in RAW264.7. Furthermore, MAPKs pathways were attenuated by SHE.

### 3.3. Toxicity of Metabolites That Exhibit Anti-Inflammatory Effects

The toxicity of metabolites on experimental models against FD particles is usually examined by in vitro lactate dehydrogenase (LDH) and 3-(4,5-dimethylthiazol-2-yl)-2,5-diphenyltetrazolium bromide (MTT) assays. The toxicity of algae metabolites tested was examined by dosing in a range of in vitro (e.g., A549 immortalized alveolar basal epithelial, RAW264.7 mouse macrophage, MLE-12 type II alveolar epithelial cell, and MH-S murine lung cells) cell lines and in vivo (e.g., zebrafish embryos, and BALB/c mice) experimental models. Fucosterol from *S. binderi* [15], DPHC from *I. okamurae* [18], dieckol and eckol from *E. cava* [17], phenolic acid from *S. horneri* [19], phlorotannins and flavonoid [21], and the ethanol extract of *S. horneri* [7] are largely non-toxic. Despite minor cytotoxic effects have been reported at concentrations of phenolic acid at ≥15.6 μg/mL [19]; DPHC at 50 µg/mL [18]; fucosterol at 100 µg/mL [15]; phlorotannins and flavonoid [21], ethanol extract [7], dieckol [17] and eckol [17] at 250 µg/mL on the tested experimental models, cytotoxicity was still well countered and more than 50% of cell viability could still be observed.

### 3.4. Mechanism of Actions of Algae to Inhibit Inflammation Caused by Particulate Matters

#### 3.4.1. Inhibition of Mitogen-Activated Protein Kinase and Nuclear Factor-Kappa B

Stimulation of TLR2 and TLR4 can lead to inflammation by PM [38,39]. Metabolites from algae can inhibit the activation of TLRs, which are involved in inflammatory responses that stimulate the inflammatory signals through the downstream upregulation of TRAF6. Consequently, algae can suppress the MAPKs and NF-κB actions by stopping the upregulation of TRAF6 that promotes pro-inflammatory gene expression and transmits external inflammatory signals to the nucleus.

**Figure 4:**
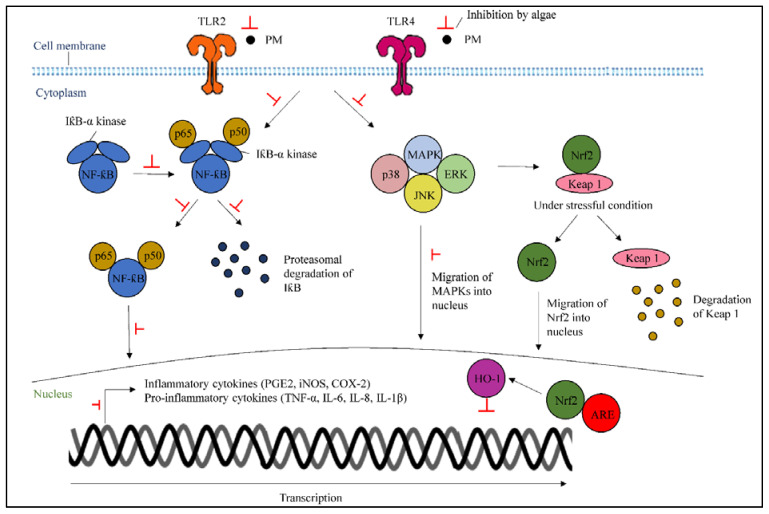
A scheme highlighting the particulate matter-stimulated inflammatory mechanisms, as well as indicating the underlying mechanisms of protective actions of algae-derived metabolites.

NF-κB represents heterodimers of both p50 and p65 subunits and locates in the cytoplasm as an inactive complex, binding to IκB-α [40]. The stimulation of the IκB-α kinase complex from LPS treatment can be inhibited by algae, resulting in the suppression of the triggered signaling pathway that also leads to the phosphorylation and activation of the MAPKs family. Algae can also inhibit the activated NF-κB, which acts as a transcription factor along with MAPKs, which trigger the COX-2, iNOS, and inflammatory cytokine production [21]. The initial degradation and ubiquitination of NF-κB, which occurs after translocation into the nucleus to initiate transcriptional processes, will be inhibited by algae treatment (Figure 4).

#### 3.4.2. Activation of Nuclear Factor Erythroid 2-Related Factor 2/Heme Oxygenase-1

HO-1 is an anti-inflammatory enzyme that reduces inflammation and is regulated by Nrf2 in the inflammatory response. In the cytoplasm, Nrf2 is bound to the Keap1 protein, which is regulated through the MAPK pathway. Keap1 initiates subsequent Nrf2 degradation under normal conditions; however, the Nrf2/Keap1 molecule is separated under stressful conditions, resulting in the initiation of nucleus translocation and the attachment of Nrf2 to antioxidant response elements (ARE) in the promotors of specific genes in the nucleus [7]. Once the attachment is completed, the activation of HO-1 transcription can inhibit inflammatory responses. Hence, algae treatment could initiate the upregulation of Nrf2/HO-1 in the PM-induced cells (Figure 4).

## 4. Limitations and Future Directions

### 4.1. Algae with Anti-Inflammatory Effect

There is a lack of study in investigating the metabolites from red and green algae inhibiting the air pollutants, as the current literature only focuses on anti-inflammatory effects of brown algae against PM-induced damage, predominantly through in vitro assays. Only four Korean brown algae species (*S. binderi*, *S. horneri, E. cava*, and *I. okamurae*) were examined against PM-induced inflammation. Generally, brown algae contribute more to anti-inflammation compared to red and green algae, due to the presence of different bioactive metabolites. However, bioactive metabolites from red algae (e.g., galactan [14], sulphated polysaccharides [13], and their solvent extracts [41]), and metabolites from chlorophyta (e.g., carotenoids [12,42], sulphated polysaccharides [14], and their solvent extracts [43]) have also exhibited anti-inflammatory effects on both in vivo and in vitro experimental models. Further studies should investigate other types of algae species in this field as there are numerous studies on the anti-inflammatory potential of algae for other diseases, but only limited studies have investigated biological potential against air pollutant-induced respiratory disorders.

### 4.2. Experimental Model

To date, most of the published studies of anti-inflammatory effects of algae against air pollutants were directed towards in vitro cell lines (RAW264.7 mouse macrophage, MH-S, A549, MLE-12, and HaCaT cells) instead of in vivo experimental models in pre-clinical studies. As this study area aims to prevent inflammation induced by PM in the lungs, cell lines such as MH-S (a murine lung cell) will be more appropriate to examine the effect in the lung cells. Sanjeewa et al. [22] made a reasonable point of using MH-S macrophage lung cells as they could not assess the anti-inflammatory effect of *S. horneri* ethanol extract (SHE) in RAW264.7 mouse macrophage against PM in their previous study. It is important to increase the applications of SHE as a functional material in clinical studies. Furthermore, anti-inflammatory effects of algae on in vitro human lung epithelial cells such as BEAS-2B cells, and/or in vivo experimental models, can be investigated as well to compare the effects and differences. Hence, future research is needed using in vitro and in vivo lung experimental models, perhaps on the same or another alga against air pollutants for respiratory diseases.

## 5. Materials and Methods

This review was accomplished according to the Preferred Reporting Items for Systematic Review and Meta-Analysis (PRISMA) Statement to report the number of accessed articles from the electronic databases (Figure 1).

### 5.1. Source and Search Strategy

The electronic literature search was conducted using Web of Science, Scopus, and PubMed on 12th of December 2020. The following search terms were used: (“alga *” OR “seaweed *” OR “microalga *” OR “marine alga *”) AND (“anti inflame *” OR “anti-inflam *” OR “anti inflame * agent *” OR “anti-inflam * agent *” OR “agent * anti inflam*” OR “agent * anti-inflam *”) AND (“fine dust *” OR “air pollution *” OR “particulate matter *” OR “air pollutant *”).

### 5.2. Inclusion and Exclusion Criteria

There was no restricted time frame for the published research articles. Duplicated articles from the three databases were removed. The included criteria were: (1) full-text research articles; (2) studies investigating the anti-inflammatory effects of algae against respiratory disorders induced by PM. In contrast, the excluded criteria were: (1) irrelevant papers; (2) languages other than English; (3) inaccessible full-text; (4) secondary source articles such as review papers, conference papers, book chapters, reports, and proceeding papers.

### 5.3. Data Extraction

Characteristics and data collected from each paper included: type of algae, species of algae, metabolites derived from algae extracts for anti-inflammatory effects against PM, toxicity of metabolites that exhibit anti-inflammatory effects, and mechanisms of actions of metabolites with anti-inflammatory properties against PM.

## 6. Conclusions

In conclusion, respiratory disorders caused by air pollutants are a serious problem and should not be neglected. As studies have shown that algae could significantly inhibit respiratory inflammation against air pollutants, people may consider having it as an alternative treatment. Therefore, the very first review in this area is prepared to provide a piece of well-summarized information about the progress of research on the anti-inflammatory potential of algae against respiratory diseases induced by air pollutants, the mechanisms of actions involved, and limitations and future directions of research. This work would assist in paving the way for a better understanding and further studies that need to be conducted, not only limited to anti-inflammatory properties of different algae on in vitro and in vivo experimental models to provide substantial evidence, but also potentially clinical trials for those promising bioactive metabolites.

## Figures and Tables

**Table 1:** Anti-inflammatory properties of algae-derived metabolites and ethanol extracts against air pollutants.

Algae-Derived Metabolites	Algae Species	Experiment Model	Mechanism of Action	Reference
Phytosterol (i) Fucosterol	*Sargassum binderi*	In vitro (A549 immortalized alveolar basal epithelial)	Reduction of apoptosis and Sub-G1 cell populations Suppression of NF-κB and MAPKs pathways	[15]
Polysaccharides (i) Alginic acid	*Sargassum horneri*	In vitro (HaCaT human keratinocyte and RAW264.7 mouse macrophage)	Suppression of NF-κB and MAPKs pathways Reduction of intracellular ROS	[16]
Polyphenol (i) Dieckol (ii) Eckol	*Ecklonia cava*	In vitro (RAW264.7 mouse macrophage)	Inhibition of inflammatory mediators and pro-inflammatory cytokines Reduction of ROS, NO production, cell death, larval mortality and blocking of larval gills in zebrafish embryo	[17]
(iii) Diphlorethohydroxycarmalol (DPHC)	*Ishige okamurae*	In vitro (HaCaT human keratinocyte and RAW264.7 mouse macrophage) and In vivo (zebrafish embryo)	Inhibition of inflammatory mediators and pro-inflammatory cytokines Reduction of ROS, NO production, cell death, larval mortality and blocking of larval gills in zebrafish embryo	[18]
(iv) Phenolic acid (gallic acid)	*Sargassum horneri*	In vitro (MLE-12 type II alveolar epithelial cell)	Attenuation of mRNA expression of TLRs, pro-inflammatory cytokines, lung epithelial cell derived-chemokines, pro-allergic cytokines TSLP and IL-33 Suppression of MAPK pathway, ERK and JNK	[19]
(v) Chromene (Mojabanchromanol)	*Sargassum horneri*	In vivo (BALB/c mice)	Attenuation of eosinophil and mast cell infiltration Reduction of IgE level Suppression of airway obstruction and mucus released from goblet cells Decreased of CD4+ and CD8+ T cell populations Inhibition of mRNA expression of RORγT and GATA3 Attenuation of phosphorylation of STAT3	[20]
(vi) Phlorotannins	*Sargassum horneri*	In vivo (BALB/c mice)	Inhibition of inflammatory mediators and pro-inflammatory cytokines Attenuation of MAPKs pathway Stimulation of Nrf2/HO-1	[21]
(vii) Flavonoid	*Sargassum horneri*	In vitro (murine MH-S cell)	Inhibition of inflammatory mediators and pro-inflammatory cytokines Attenuation of NF-κB and MAPKs pathways Inhibition of upregulated mRNA expression levels of toll-like receptors	[22]
Ethanol extract	*Sargassum horneri*	In vitro (RAW264.7 mouse macrophage)	Attenuation of MAPKs pathway Stimulation of Nrf2/HO-1	[7]
